# Uncertainty of Reported Behavior Dynamics and Its Relationship to Socio-Political Ideologies and Affiliation

**DOI:** 10.3390/e28050545

**Published:** 2026-05-11

**Authors:** Patric C. Nordbeck, Christine Shi, Anjali Dutt

**Affiliations:** 1 Department of Psychology, Lund University, 22100 Lund, Sweden; 2 Department of Psychology, University of Cincinnati, Cincinnati, OH 45221, USA

**Keywords:** politics, neoliberalism, Islamophobia, surveys, Shannon information, entropy, immigration, perception

## Abstract

People’s willingness to include others and their level of suspicion of those perceived to not belong are constrained by political affiliation and adherence to ideologies such as neoliberalism, Islamophobia, and ethnocentrism. In an era of heightened polarizing discourse about immigrants, the interaction between changing information and constraints can be leveraged to understand how dynamic narratives affect inclusory and exclusory behaviors. This study provides a combination of time-series generating methods and the survey approach to situate participants in a developing scenario. Eighty-two participants completed the dynamically modified survey in a scenario involving an immigrant family moving in next door and responded to two affordances: perceived invitability and reportability of the family. Participants’ responses to each of twelve new situations formed time series of changes in reported inclusion and exclusion. Shannon information was used to quantify the amount of information (or uncertainty) that any given instance in the data series represents about the set of reported behaviors. The results showed clustering around adherence to neoliberal ideology, Islamophobia, ethnocentrism, and political affiliation, along with their relation to uncertainty of the time series. We discuss potential implications for perceptions of others’ behavior and the potential of the modified survey and affordance-focus as dynamic and relational methods.

## 1. Introduction

The process of forced migration to a new continent, country, culture, and community is enveloped in many different kinds of uncertainties. It can for example be difficult to know who perceives you with enough suspicion that even benign behaviors can lead to direct confrontation or unnecessary law enforcement involvement. From the perspective of a citizen there are many influences that affect how immigrants are perceived. For example, both ideological commitments and political affiliation interact with day-to-day experiences (e.g., in relation to threat perception [1,2]), but in experimental research this interaction is not given much attention [3]. And when empirical research is produced they tend to employ cross-sectional design and compare average changes [4,5], or generalize from economic games [6]. There is consequently little research attempting to capture the (potentially) dynamic interaction process with exposure to everyday situations [7]. This is important since it has the potential to better account for real life situations and thus increase ecological validity. To fill this gap in the current study we combine principles from Dynamic Systems Theory [8] and Ecological Psychology [9,10,11,12] with the survey method and concept of ideology as global constraint on behavior [6,13] to create a dynamic experimental context. By exposing participants to an everyday scenario with subsequent, additional everyday situations, potential changes in participant reactions can be measured on relevant dimensions like inclusion and exclusion. Finally, we argue that the collective sets of reported behaviors are representatively quantified by Shannon information, linked to ideologies and political affiliation, and generalizes well to real life experiences.

Given the complexity and multiplicity of circulating socio-political narratives, there is growing need for social science approaches to assess the dynamic nature of shifting information and portrayals of contemporary social issues [14]. There are multiple ideological factors that affect the perception of immigrants, and in particular, immigrants from the Global South [15,16]. According to the United Nations, in 2024 there were 304 million people living in a country other than their countries of birth and numbers of global migrants are expected to grow due to increasing numbers of refugees and asylum seekers, changes that arise from shifting labor forces, and factors such as climate change that impact where people can safely live. The United States continues to have the largest number of immigrants in the world, with 19% of the global migrant population. In the current study we investigate how multiple ideological factors and shifting information impact reactions towards immigrants with a United States sample.

Explicitly espoused ideological factors and narratives by political parties are not the only influence on individuals’ political perceptions [17]. Ideologies are here operationalized as constraints (restrictions) on perceptions of (and actions against) people that the particular ideology is about [6]. Interestingly, research on ideological alignment (that certain beliefs show strong interconnections) shows that people who affiliate with more left politics and parties have stronger alignment in beliefs compared to the right [18]. Adherence to ideologies can thus be said to constrain one’s interactions with dominant societal narratives since they are latently woven into political discourse and thus affect individuals’ perceptions of contemporary social issues [19]. For example, in the United States, neoliberalism is increasingly described as a dominant ideology shaping numerous aspects of US society. Neoliberal ideology is rooted in the belief that competition, individual responsibility, and limited state intervention should be the primary means of organizing social life and human wellbeing [20]. While the term has been adopted widely, there is some discrepancy in how it is defined and researched depending on discipline and research aims [21]. Here, we measure it at individual level by quantifying how strong adherence is to various parts of its principles, although originally it is rooted in economic terms through capitalist beliefs connected to market freedom and minimal state interference (except to uphold capitalist motives such as private property rights). However, despite wide use in research, neoliberalism is uncommonly claimed as the basis of political platforms by politicians, and lay individuals rarely explicitly describe neoliberal beliefs as shaping their perspectives on social issues. Nevertheless, a growing body of research documents how neoliberal ideology are related to individuals’ perceptions on a number of social issues and personal experiences [22]. Similarly, narratives about American exceptionalism influences numerous institutions in the US that constrain attitudes and perspectives of citizens, and is evidenced in ethnocentric beliefs [23] (i.e., the belief that one’s own culture is superior to all others [24]).

Political narratives regarding immigrants are often laden with images of risks and gains. For example, conservative politicians in the United States and Europe have argued for restricting migrants from Muslim majority countries by associating migrants with images of terrorists [25]. Attitudes towards Muslim immigrants are particularly worth examining in the United States context due to ongoing political controversy related to immigrants from Muslim majority countries. For example, during his first term as president, Trump banned immigrants from seven such countries. Additionally, those advocating acceptance of migrants will discuss economic gains that come from welcoming immigrant groups, as well as the benefits that arise from more diversity in workplaces and communities more broadly [26]. There are also moral narratives people encounter and relate to about “welcoming the stranger” and offering refuge to those who have experienced hardship in both religious and national contexts. Because individuals likely encounter multiple narratives about immigrants, it is possible that when interacting with immigrants, various ideologies and values impact how sensitive people are to potential risks [27]. Using methods that produce time-series data therefore may yield insights into how interaction with changing information can guide shifts from inclusion to exclusion of immigrants. For example, someone more fearful of others would likely stop including if their perception of the individual or group becomes seen as a risk [28]. In turn, from the perspective of the immigrant experiencing shifting inclusion, this can be perceived as uncertainty as to whether or not they can trust the other to act in their best interests. Furthermore, utilizing methods that allow for more nuanced analysis of the factors that shape (reported) behaviors toward immigrants may be particularly helpful in understanding ideological forces (i.e., neoliberalism, ethnocentrism, and Islamophobia) that are highly prevalent, but less well understood among both psychologists and lay populations.

The spread of technologies, social media, and other forms of communication have enabled global access to various political and moral narratives [29]. Although there has never been a time in which social issues and political narratives have been static, the contemporary era brings a heightened degree of news about both present and impending sociopolitical turmoil [29]. Additionally, increasing levels of political polarization in many societies suggest stricter adherence to specific ideological narratives [30], where party narratives alter in accordance with opportunistic political aims [31]. For example, political narratives about immigrants and refugees shift depending on migrants’ country of origin, along with attitudes towards these groups [32]. In turn, these shifts are often rewarded by party followers in election cycles [33]. Research on political affiliation in this context suggests a dynamic relationship between party preference and attitudes towards migrants. For example, longitudinal research documents that as individuals shift their political affiliation more to the right, they tend to become stricter about immigration, and as they shift to the left they become more supportive of pro-immigrant legislation [34]. Research in the United States context also suggests that in addition to consideration of economic and cultural factors, citizens perspectives on immigrants become more positive when they perceive them as political allies (i.e., those anticipated to belong to the same political party [35]).

### 1.1. Socio-Political-Psychological Ideologies and Reactions to Immigrants

Neoliberalism is the dominant ideological force shaping ideologies and identities in the United States [22,36,37,38,39]. Beginning as a set of political-economic theories and practices, neoliberalism is now accepted as a dominant ideological basis for common sense. At its origin, neoliberalism posits that human well-being and social good is best advanced through the strict liberation of individual market freedoms and reduction of state interference to a bare minimum, except to uphold individual freedoms connected to capitalist motives (e.g., private property rights, financial capital). In every-day practice, neoliberalism prescribes free market solutions and the gospel of personal responsibility to nearly every social, political, economic, and personal issue and decision [36,37]. Although there is an abundance of research connecting neoliberal policies to increased inequity [40], neoliberal beliefs justify this inequity as beneficial in inspiring personal agency and fostering the development of the entrepreneurial self, innovation, and wealth creation [39]. Neoliberalism is also associated with the denial of sexism, racism, and other institutional discrimination and prejudice and the reality of a post-racial and post-discrimination society [41]. While the literature on neoliberalism’s impact on our perceptions of others is quickly growing, we lack an understanding of its potentially dynamic impact on behavioral responses. For instance, endorsement of neoliberal ideology is positively associated with social dominance orientation [22] and the belief that misfortune and failure are attributed to personal inadequacies (rather than structural injustices [41]). And internationally, anti-migrant sentiment and policies have also been linked to neoliberal values and aims [15,42,43,44]. However, in an interpersonal context involving migrants of Color, the question remains: Does neoliberal ideology individually constrain behavioral responses related to repeated day-to-day interaction when juxtaposed with other social factors such as the desire to be friendly (and inclusive) and to feel safe (and be exclusive)?

Ethnocentrism is a nearly universally entrenched pattern of discriminatory behaviors and attitudes which include viewing one’s own culture, values, and standards as universal and others’ as inferior and deserving of moral exclusion (i.e., being excluded from the ingroup’s scope of justice, thus often treated with double standards [45,46,47]). Connections between ethnocentrism and anti-migrant sentiment are abundant [48,49,50]. In addition, ethnocentrism has been linked to an absence of cooperative relations with out-groups and is strongly related to ethnic conflict [51,52], and consumer behavior [53]. Building on its ubiquity and connections found between ethnocentrism and moral exclusion [15], we are interested here to understand it’s relation to perceptions and actions toward others as well as to other ideologies.

Islamophobia is generally described as discriminatory attitudes and behaviors directed at Islam, Muslims, and Arabs in Western cultures which have long dominated media and sentiment related to these groups [54,55]. Islamophobia has also been described as cultural racism with the basis of race as a social construct [56]. Islamophobia’s link to neoliberalism and their interconnected contribution to racialized governmentality in the West is only beginning to be understood critically [57]. Yet, it’s impact on behavior and attitudes have been well established to lead to discrimination and harm on nearly all fronts, globally, communally, and individually [58,59,60,61,62]. Internationally widespread Islamophobia impacts perceptions and behaviors and must also be considered when investigating behavioral responses related to migrants of Color.

Although higher levels of neoliberal ideology, ethnocentrism, and Islamophobia tend to be associated with right-wing political affiliation in the United States [63], the prevalence of narratives that reflect these ideologies at multiple ecological levels renders them particularly meaningful in assessing reactions towards immigrants dynamically. Additionally, it could be argued that neoliberalism should be inversely related to ethnocentrism and Islamophobia due to the central belief of neoliberalism that performance of individual rather than their affiliations (national, religious, political, etc.) should determine success [15]. Nevertheless, survey research consistently documents positive correlations between neoliberal ideology and ethnocentrism or similar constructs related to beliefs about other cultures [64]. This seeming contradiction further encourages research into the dynamic nature of ideological beliefs in realistic situations that involve the inclusion or exclusion of migrants.

### 1.2. Dynamic Measurement and Shannon Information

It is well understood that the political sphere and individual psychology shape one another [65]. Through iterative, dynamic processes individuals encounter political narratives and institutions that influence their attitudes, beliefs, and reactions towards events that happen in their environments [66]. In turn, individuals take these perspectives and incorporate them in the formation of political structures and platforms. Although few social or political psychologists would deny the dynamic nature of political and psychological attitude development, measures used to gauge them tend to capture only momentary snapshots, correlations, or compare averages - not changes over time, as new information unfolds [7]. In several fields of research, it is becoming common to emphasize the importance of theory, methods, and analyses that carry through the dynamic nature of a phenomena of interest (e.g., in sports pedagogy and physiotherapy [67,68], social psychology [7], resilience [14], and in creativity and aesthetics [69]). This inspired the present project to include the (otherwise most commonly statically used) survey method combined with a scenario that develops in several steps and responses are measured at each one.

In decision making research there are many examples of employing more dynamic methods during measurement (e.g., [28]) inspired by a dynamical systems framework [8,70,71]. Under this framework the central interest lies in observing how a system or individual’s behavior unfolds and changes over time (whileout of scope to delve deeper into here, those interested should explore Dynamic Systems Theory [8] and complex systems [72] further), and not necessarily only what an average behavior of a group is pre- vs. post-intervention [73,74]. Once we have access to measuring behavioral states and their changes over time, they can be sued for various kinds of dynamic patterns. Here, Information Theory [75,76,77] is one such theory that can describe the collective behavioral state change patterns in meaningful ways, albeit still linearly and additively (see examples in [78]). Shannon information relates to the probability of a certain state or outcome, where an observed state with 100% probability is ‘not surprising’ and the less probable the observed state, the more ‘surprising’ it is [79]. These ideas are related to the concept of entropy (in Information Theory) where the more information an observed state reveals about the larger system the lower the entropy [78]. A physical example of this is the arrangement (or configuration) of molecules in the different states that H_2_O can be in: as a solid, the arrangement of molecules are in a strict lattice with little possibility of movement (or reconfiguration); as liquid, its molecules are more free to move around and thus more arrangements are possible; and in gaseous form, molecules can move about quite freely resulting in many different arrangements. Taking a snapshot of the molecular structure of ice thus means we can “know” a lot about the whole lattice and also over time (ceteris paribus), uncertainty is low. In gaseous form the molecular structure may only be in that particular arrangement for that snapshot of time, so one measurement does not reduce uncertainty to the same degree. As Hartnett [80] succinctly puts it: “acloud has higher entropy than an ice cube” (para. 9).

Shannonhimself [81] and others [82] warn about stretching Shannon information to explain things it did not intend to explain. Chomsky [82] exemplified how its usage can be problematic from a linguistics perspective by applying the concept to sentences that are grammatically correct but that do not provide any actionable information: “colorless green ideas sleep furiously” (p. 15). That is, if we analyze the sentence syntactically, it would yield low entropy and we might make the mistake of interpreting this as also representing a lot of information. In essence, there is a structure-content mismatch. We apply it here to a series of changes in binary responses and interpret the values of Shannon information (*H*) as indicators of how much can be known about the set of changes in the response-series. That is, given a series of changes in binary responses, does one such change inform about that person’s other responses? Also, if the uncertainty of how changeable someone’s responses are relate to (more traditional) measures like socio-psychological ideological and political scales, this bears on uncertainty being able to be predicted by ideological and political scales in the US context, and what potential implications could be drawn about citizens’ reported behaviors on this basis.

Shannon information and information theory treats communication as a mathematical and probabilistic process of encoding and decoding information, or in brevity: information processing [83]. Mentioned earlier, it does not differentiate between semantical meanings, but is concerned about probabilities of occurrences within a message. The less frequent, the higher its information content. This means however that it is not obvious how Shannon information could be used for psychological phenomena beyond the particular neuropsychological perspective that assumes the brain to be a biological information processing device [84]. There is, for example, research attempting to build models that the brain (presumably) holds and uses in the context of aesthetic preferences [85]. However, although being about aesthetics, only theoretical assumptions are made about what is aesthetically pleasing or not. Actual measures of aesthetic judgments from participants are not used but inferred from indirect measures of image-related properties like chromatic complexity or resolution [86]. Exceptions can be found, for example research on the etiology of rumors in social media [87], or in highly constrained laboratory tasks like relating visual ambiguity of a stimulus to reaction times in priming studies [88]. It is also common that even if the aim is to explain a behavior, the underlying process to be understood is information propagation [89], as opposed to the behavior itself. Much research has treated uncertainty in non-social contexts and it is rare that the focus is on behaviors in relation to other human beings (although see [90] for an example on uncertainty related anxiety or [91] for social uncertainty). This is an empirical gap we contribute to filling with this study.

### 1.3. Aims, Hypotheses, and Current Study

The main aim is to leverage changing social information to ‘push’ participants’ perception of a hypothetical immigrant family, such that reported inclusion and exclusion varies over the added situations. More specifically, we explore the relationships between ideologies, political affiliation, uncertainty (Shannon information), and the propensity to report inclusion and exclusion of the hypothetical family. As presented in Section 1.2, previous research suggests that the three ideological scales used here should correlate positively with each other and with more conservatively leaning political affiliation. Based on this, the first hypothesis is that participants cluster around adherence to the three ideologies and political affiliation, such that higher adherence and ‘more conservative’ go together (and vice versa). Also, less conservative should lead to more constrained reported behaviors, as less conservative individuals have been linked to less variable behavior (i.e., more ideologically aligned). Therefore the second hypothesis is that the clusters relate to Shannon information (based on the dynamic, reported, behavioral time series) such that lower adherenceand less conservative also show lower uncertainty (higher alignment). We will also test if the same relation holds for both reported inviting (inclusion) and reporting (exclusion).

In order to test the hypotheses a scenario was created to situate participants in the context of responding to information about an immigrant family who have just moved into their suburban community. Participants were asked to make decisions about two affordances (here defined simply as action possibilities [10,11]), (a) invitability; if they would invite the family to an upcoming potluck event, and (b) reportability; if they would report the family to the local neighborhood watch group. Then, we continued the narrative of the scenario through twelve additional situations that were serially presented containing descriptions of what the neighbors were doing. Comfortableness was varied in the series of situations and reported behavior was recorded through Yes/No responses to inviting and reporting the family after each situation. Descriptive data, visualisations, K-Means Cluster analysis, and ANOVAs were then used to test the hypotheses and explore the data.

## 2. Materials and Methods

### 2.1. Participants

One-hundred participants were recruited via Prolific Academic with the inclusion criteria of being US citizens over the age of 18 and currently residing in the US. Participants were paid $12 for completing the study. Eighteen responses were identified as automated (see Section 2.2.5 for details) resulting in a final sample size of 82 participants. Their age ranged between 18 and 65 (*M* = 34.89, *SD* = 9.46) and 38 identified as women, 39 as men, four as nonbinary, and one did not respond. For ethnicity/race, one participant responded Hispanic American, four Asian American, three mixed, 19 Black American, and 55 White American. On party affiliation, one participant responded that they were Libertarian, one Independent, 33 Democrat, 14 Republican, and 33 responded that they were not affiliated with any particular party.

### 2.2. Materials, Task, and Procedure

**Figure A1:**
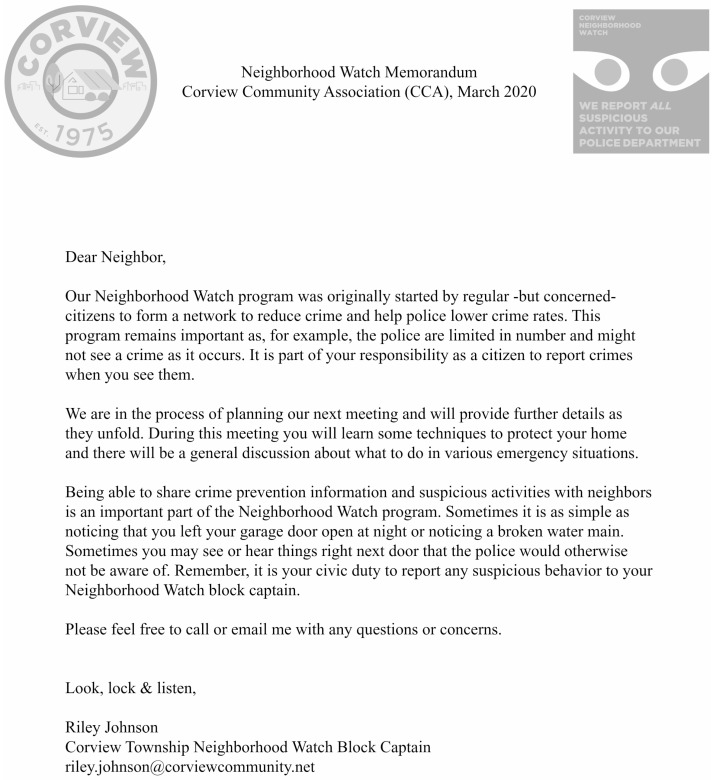
Neighborhood Watch Memorandum.

**Figure A2:**
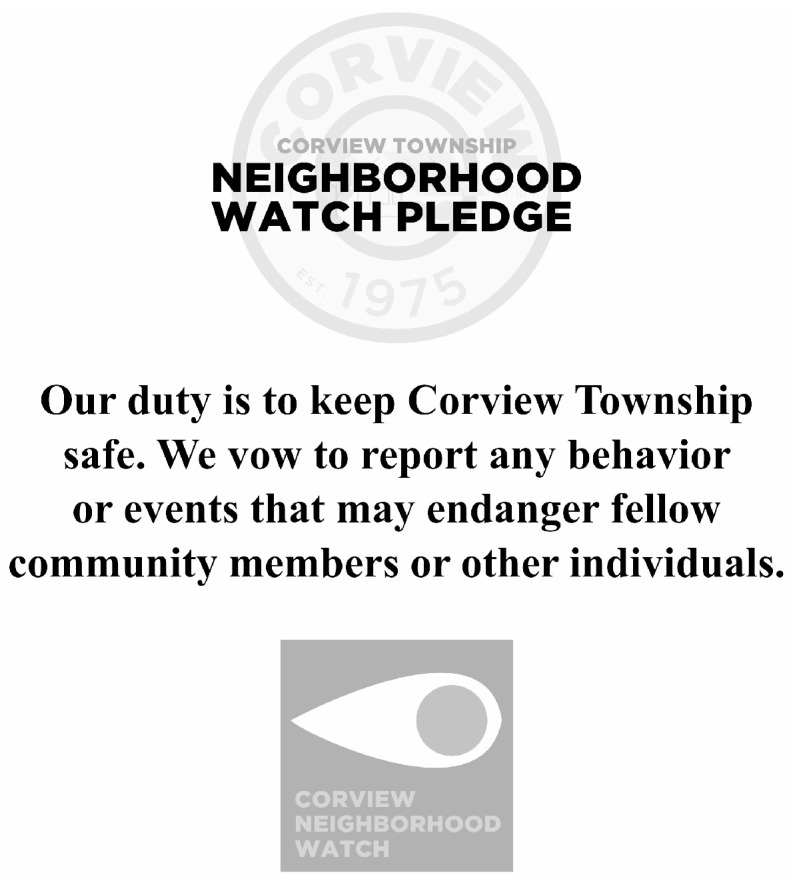
Neighborhood Watch Pledge Note.

**Figure A3:**
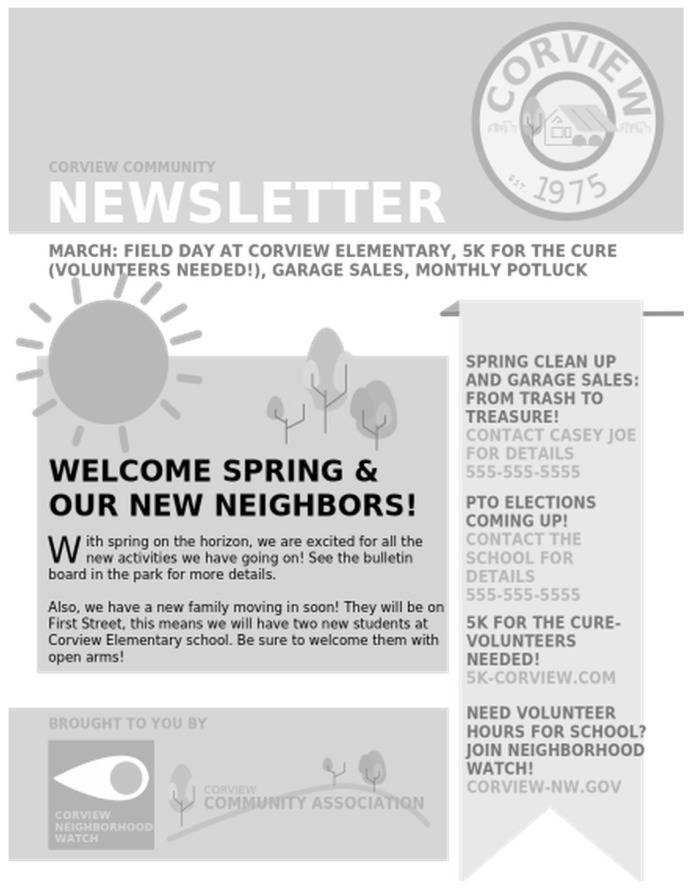
Corview Community Newsletter.

**Figure A4:**
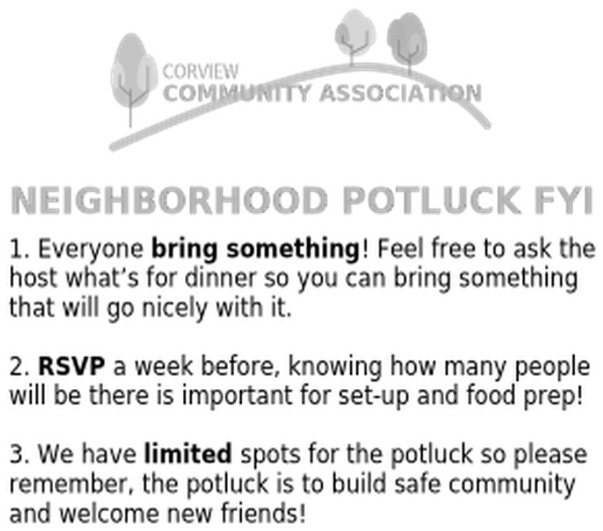
Pot Luck Note.

The study was structured into three general parts: (1) scenario background (full materials can be found in Appendix A, Figure A1, Figure A2, Figure A3 and Figure A4), (2) the dynamic narrative portion containing 12 discrete situations (Appendix B), and (3) socio-political scales and demographic questions.

#### 2.2.1. Scenario Background

The study scenario was set in ’Corview Township’, a fictional neighborhood in the United States. Participants were asked to imagine that they were a resident of this neighborhood and that a family has recently moved in next door. Four background materials situated the participants in the scenario: a letter and a pledge note from the local neighborhood watch group as well as a newsletter and a potluck reminder from the local neighborhood association. All materials related to the neighborhood watch group were based on the National Neighborhood Watch Program and its website [92].

The scenario background introduces two types of response items to participants: “Do you invite the family to the potluck event?” (invitability) and “Do you report the family to the neighborhood watch group?” (reportability). Participants could respond Yes or No along with their confidence in their decision and an optional open-text response. Participants responded to these questions both before and during the dynamic narrative portion, the former are referred to here as initial response items. Only Yes/No responses are reported and used in the analyses.

#### 2.2.2. Dynamic Narrative

The dynamic narrative introduces new (yet ordinary and benign) information about the neighboring family, simulating realistic day-to-day observations. The twelve situations were created with three categories in mind: four relatively comfortable situations (e.g., “A couple of days later, while walking to a neighborhood activity, you see the kids outside playing hopscotch and laughing with some older kids while they’re playing word games”), four neutral situations (e.g., “The next day, you see the kids playing outside and in your conversation with them, they mention they’re from Syria. A few moments later the mother rushes out of the house and hurries the kids in, barely making eye contact with you”), and four uncomfortable situations (e.g., “On a late night walk around the block, you hear adults and children yelling from the new neighbor’s house”). After each situation participants re-answer the two affordance questions, resulting in two time series of 12 Yes/No responses each (referred to as narrative response items).

#### 2.2.3. Scales and Demographics

Endorsement of neoliberal ideology was assessed using the 25-item Neoliberal Beliefs Inventory [41]. A sample item is “right now, pretty much all Americans are free to live any kind of life they want”. Participants indicated their degree of agreement with each item on a five-point Likert scale ranging from agree to disagree. Summed scores were computed, with higher scores reflecting greater adherence to neoliberal ideology (minimum score = 25, maximum = 125). Reliability analysis conducted using MacDonald’s *ω* [93] was *ω* ≈ 0.96, no item could be removed to improve the scale.

Twenty-five items including 16 items from the Islamophobia scale (e.g., “the religion of Islam supports acts of violence”; [94]) as well as 9 items from the feelings of threat and destructive ideologies subscale of the Moral Exclusion scale [45] modified to specify Muslim immigrants (e.g., “the presence of Muslim immigrants makes our future more uncertain”) were used to assess the degree to which participants held negative attitudes towards Muslim immigrants. Participants indicated their degree of agreement with each item on a seven-point likert scale (minimum score = 25, maximum = 175). All but four items on the scale were reversed so that higher numbers indicated higher adherence to Islamophobia (ω ≈ 0.98), no item could be removed to improve the scale.

Sixteen items measured the extent to which participants believed that the United States was superior to all other countries [47]. Six items were reversed so that a higher score on the scale indicates higher ethnocentrism. A sample item is “most other cultures are backward compared to my culture”. Participants indicated their degree of agreement with each item on a five-point scale ranging from agree to disagree (minimum score = 16, maximum = 80). Reliability was ω ≈ 0.92, no item could be removed to improve the scale.

Four demographic questions were asked; Age, Gender, Ethnicity, and Party Affiliation, all as write-in answers. Also a fifth question asked participants to rate their Political Affiliation on a scale from 0–10 where 0 was labeled “progressive”, “liberal” was placed between 2 and 3, “moderate” was placed at 5, “conservative” was placed between 7 and 8, and 10 was labeled “very conservative”.

#### 2.2.4. Procedure

On the first page of the survey, participants were informed about their right to withdraw consent at any time and voluntary participation in the experiment. They provided informed consent digitally and were then given a general outline of the study and that they should feel free to answer based on their personal reactions to the content. The scenario background materials were then presented, situating the participant within the neighborhood with a spouse and two young children. Participants then responded serially to the first six situations, answered an awareness check question, and continued on to the last six situations and responded to the narrative response items after each situation. They then answered the items for the three scales, demographic and political affiliation questions, and were finally thanked for their participation.

#### 2.2.5. Data Preparation

The data was analyzed both at whole dataset level as well as based on subgroups identified by a K-Means Cluster analysis. The three ideological scales along with Political Affiliation was submitted to the K-Means Cluster analysis (using the snowCluster module in jamovi [95,96]).

##### Optimal Cluster Size Determination

The number of clusters to set for the K-Means Cluster analysis was estimated through the Matlab 2024b function *evalclusters()* with three different criteria; Calinski-Harabasz [97], Davies-Bouldin [98], and the gap statistic [99]. The latter method was additionally run with four different distance metrics; squared Euclidian distance, sum of absolute differences, one minus the cosine of the included angle between points, and one minus the sample correlation between points. The Calinski-Harabasz and Davies-Bouldin criteria both estimated the optimal cluster size to be two clusters, whereas half each of the four gap statistic analyses showed a higher gap statistic for two and three clusters respectively (the difference in gap statistic was higher than the standard error for each analysis). Due to the mixed results we decided to use three clusters based on the additional nuance it provided while preserving the differences that were found in the two-cluster analysis. Each participant’s cluster belonging was saved for later analyses and each cluster is described in larger detail in Section 3.

##### Calculation of Shannon Information

Each participant’s two time series of 12 Yes/No responses were coded “1” for Yes and “0” for No, and then the difference between each consecutive data point was calculated:

(1) $$ts_{diff}=ts_{i}-ts_{i-1}.$$

The differentiated time series were then applied to the calculation of Shannon information with the equation

(2) $$H\left(X\right)=-\sum (P\left(x_{i}\right)\times log_{b}\times P\left(x_{i}\right)$$

where *H* is the Shannon information of the variable *X*, *n* are the total possible outcomes, *P(x_i_)* is the probability of the *i*th outcome, and *b* is the base logarithm (here *b* = 2, or *bits*).

### 2.3. Note on Use of GenAI

No GenAI was used for any part of preparing this manuscript nor for any aspect of the research or analyses.

## 3. Results

### 3.1. Base Tendencies and Range of Reported Inviting and Not Reporting

Before turning to the hypothesis driven analyses, it is worth noting the results of the initial response items to inviting and reporting the family. The ratio of Yes-to-No responses (where 1 = 100% Yes and 0 = 100% No) to invitability and reportability was 0.9 (CI^95%^ = [0.84 0.97]) and 0.34 (CI^95%^ = [0.24 0.45]) respectively. The variables were analyzed with Wilcoxon rank one-sample *W*-tests (since the normality assumption was violated) with a non-directional comparison to chance level (0.5). Both tests were significant, *W* = 3071 and 1162 respectively, both *p* < 0.01, rank biserial correlation = 0.81 and −0.32. The effect of the baseline scenario materials therefore was for participants to tend to invite and not report the neighbors, with a strong tendency to invite and a medium tendency to not invite.

**Figure 1:**
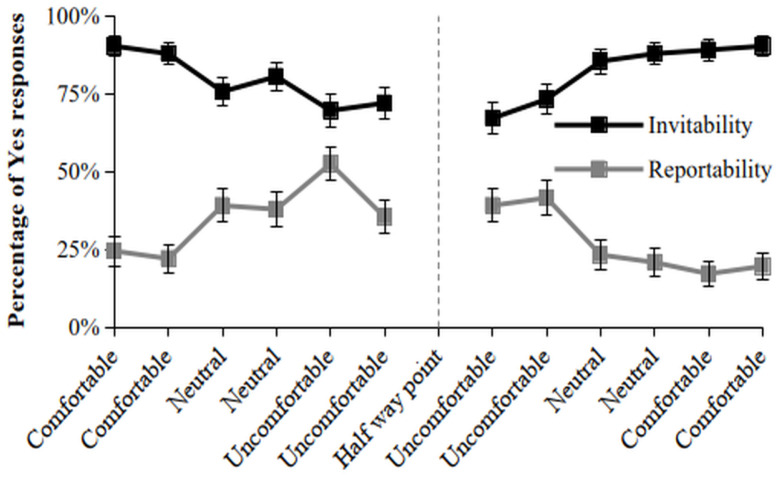
Percentage of Yes Responses (error bars reflect standard error) for the Narrative Response Items for each Affordance.

If participants would not change their decisions at all during the dynamic narrative portion, then the response ratio should not fluctuate beyond the confidence intervals of the initial response items. As seen in Figure 1, the ratio for both invitability and reportability change across the narrative response items with a general trend that Yes responses first decrease for invitability and then increase in the second series again (range = 67–90%). The pattern for reportability is inverse to invitability with a range of 17–52%. Thus, invitability varies beyond the lower bound of the initial response item and reportability varies beyond both the lower and higher bounds, indicating state shifts at whole-group level.

### 3.2. Hypothesis 1: Ideological-Political Clustering

**Figure 2:**
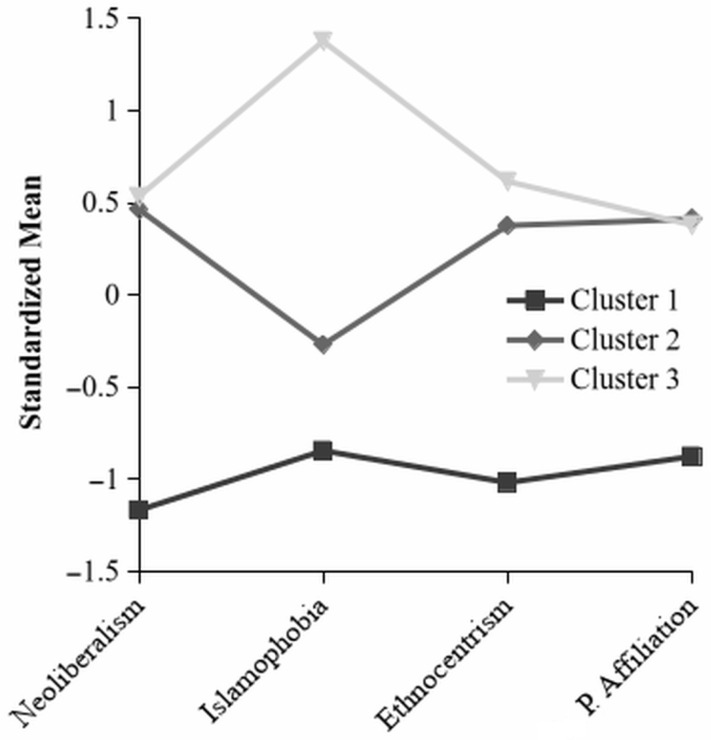
Standardized Cluster Centroids by Ideological Scale and Political Affiliation.

The K-Means Cluster analysis was conducted using the Hartigan-Wong algorithm, 10 random starting values, and *k* = 3 clusters. The total amount of within cluster variation was 140.6 and the between cluster variation was 173.6. Participants were divided into the clusters such that 24 were assigned Cluster 1, 35 were assigned Cluster 2, and 22 were assigned Cluster 3. The standardized centroids of the clusters are reproduced in Figure 2 below (unstandardized means and standard deviations can be found in Table 1), showing that Cluster 1 is low on all ideological scales (neoliberalism = −1.17, Islamophobia = −0.85, ethnocentrism = −1.02) and on Political Affiliation (−0.88), and Cluster 2 and 3 are relatively high on adherence to neoliberalism (0.47 and 0.53), ethnocentrism (0.38 and 0.62), and Political Affiliation (0.41 and 0.38), but on Islamophobia Cluster 2 (−0.27) is lower than Cluster 3 (1.38).

Cluster 1 represents a group of participants that report low adherence to all of the scales and also report being more politically left than Liberal, including participants responding Progressive and left of Liberal. Cluster 2 consists of participants reporting high adherence to neoliberalism and ethnocentrism, but medium-low adherence to Islamophobia, and report being Liberal and more politically right than Liberal (e.g., Conservative and Very Conservative). Finally, participants in Cluster 3 report high adherence to all scales, and on Political Affiliation report being Liberal and more politically right than Liberal (similarly to Cluster 2).

To confirm that the clusters are statistically different from each other, a non-parametric (Islamophobia did not meet normality or homogeneity assumptions) Kruskal-Wallis one-way ANOVA was performed with the three scales and Political Affiliation as dependent variables and Cluster as a grouping variable. All four variables were significant (all *p* < 0.001, η^2^ for neoliberalism = 0.56, Islamophobia = 0.75, ethnocentrism = 0.5, and Political Affiliation = 0.37). Dwass-Steel-Critchlow-Fligner pairwise comparisons revealed that all differences between Cluster 1 and Cluster 2, and Cluster 1 and Cluster 3 were significant (all *p* < 0.001), but only the difference in Islamophobia was significant between Cluster 2 and 3 (*p* < 0.001, all other Cluster 2 to 3 comparisons *p* > 0.05). Perhaps unsurprisingly, the main difference to a two cluster K-Means Cluster analysis was that almost all Cluster 2 and Cluster 3 participants were put in one cluster.

### 3.3. Statistics of Narrative Response Items by Cluster Belonging

**Figure 3:**
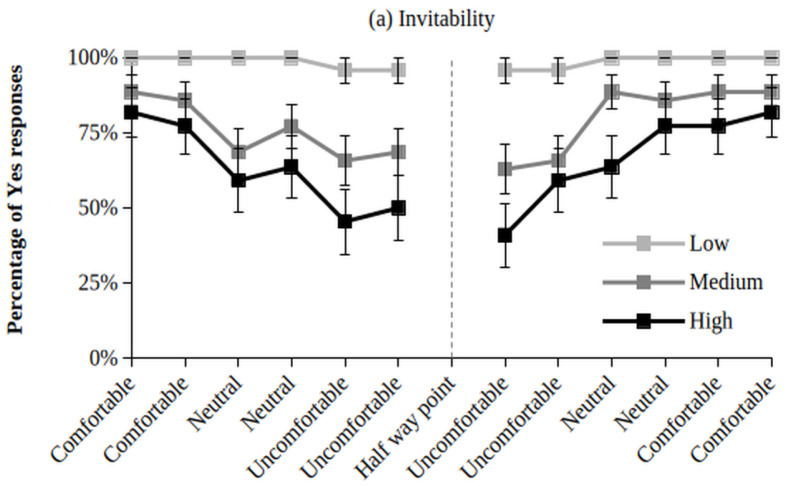

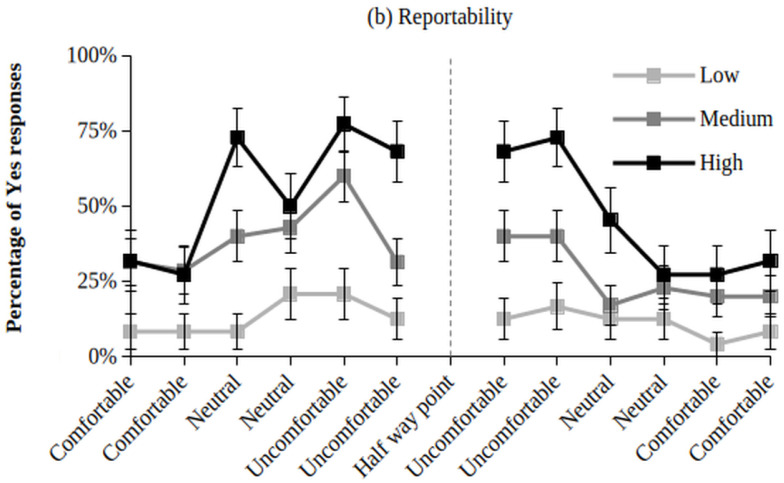
Percentage of Yes Responses (error bars reflect standard error) for the Narrative Response Items for each Cluster by (**a**) Invitability and (**b**) Reportability.

Once cluster belonging was determined, the descriptives for Yes-to-No ratio by whole group (Section 3.1 above, Figure 1) were divided by cluster and re-visualized to show cluster variation in inviting and reporting (see Figure 3). Cluster belonging shows clearer trends between Cluster 1 and both Cluster 2 and 3 than between Cluster 2 and 3 on both reported affordances. Cluster 1 tends to invite and not report whereas Cluster 2 and 3 tends to stop inviting (and then begin re-inviting again) and start reporting (and then stop reporting again) when comfortableness is lower (and then higher).

### 3.4. Hypothesis 2: Differentiation of Shannon Information Based on Cluster Belonging

**Figure 4:**
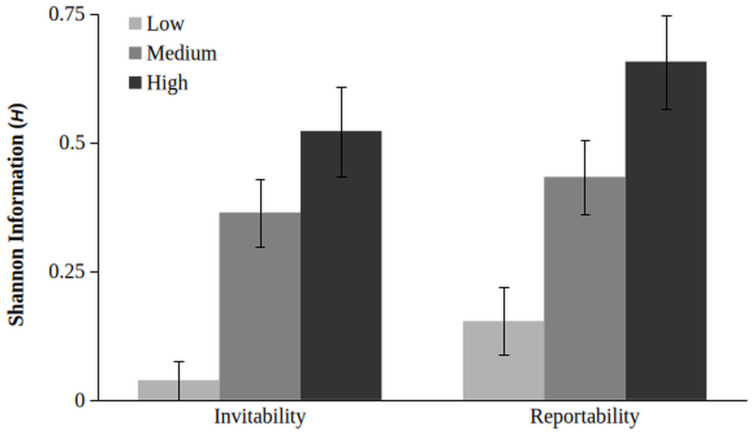
Mean Shannon Information for each Cluster and Affordance (error bars reflect standard error).

The clusters were used as a grouping variable to probe for patterns in the Yes/No change time series as quantified by Shannon information (*H*), both for invitability (or inclusion) as well as reportability (or exclusion; see Figure 4 for a visual overview of descriptive statistics). For invitability, Cluster 1 had a low mean of *H* = 0.04 (*SE* = 0.04), Cluster 2 had higher mean of *H* = 0.55 (*SE* = 0.1), and Cluster 3 had the highest mean of *H* = 0.68 (*SE* = 0.12). This pattern repeats for reportability; Cluster 1 *H* = 0.19 (*SE* = 0.08), Cluster 2 *H* = 0.58 (*SE* = 0.09), and Cluster 3 *H* = 0.75 (*SE* = 0.12).

To test whether cluster belonging differentiates the groups on Shannon information (*H*) a non-parametric (neither variable met normality or homogeneity assumptions) Kruskal-Wallis one-way ANOVA was conducted with Shannon information for invitability (*H_inv._*) and reportability (*H_rep._*) as dependent variables and Cluster as grouping variable (Cluster 1, 2, and 3). The omnibus ANOVAs were significant for both (χ^2^_inv._(2) = 19.6, χ^2^_rep._(2) = 15.2, both *p* < 0.001, ϵ*^2^* for *H_inv._* = 0.25 and *H_rep._* = 0.19). The Dwass-Steel-Critchlow-Fligner pairwise comparisons showed significant differences between Cluster 1 and 2, and Cluster 1 and 3 within both dependent variables (*p* ≤ 0.02), but no significant differences between Cluster 2 and 3 were found (*p* = 0.29 for *H_inv._* and *p* = 0.13 for *H_rep._*).

## 4. Discussion

Narratives about immigrants come in dynamic forms in the real world and interact with people’s perceptions, ideologies, and relations to the target group. In turn, perceiving people’s decisions and behaviors can invite uncertainty (in the informational sense) from an observer if their inclusion or exclusion changes over time. A modified survey method with a dynamic narrative (and repeated questions) was used to investigate the connection between common ideologies, political affiliation, and uncertainty (as measured by Shannon information). Participants were situated in the hypothetical “Corview Township” where an immigrant family moves in next door. Twelve new situations describing behaviors of the family were presented serially and participants responded to two questions about perceived affordances (invitability and reportability of the family) after each situation, forming two time series of Yes/No responses. The first hypothesis was that a K-Means Cluster analysis would group participants according to their adherence to three ideological scales (neoliberalism, Islamophobia, and ethnocentrism) and Political Affiliation. The second hypothesis was if cluster belonging could predict uncertainty as applied to the Yes/No change time series. The first hypothesis was supported in that clustering occurred in Cluster 1 around low adherence to the ideologies and more leftist political affiliation, Cluster 2 and 3 adhered moderately and highly to Islamophobia, and both clusters had high adherence and liberal-conservative leaning (i.e., more right). The second hypothesis was partially supported as a non-parametric one-way ANOVA showed that Cluster 1 differed significantly from Cluster 2 and 3 on uncertainty for both invitability and reportability but Cluster 2 and 3 did not differ on either. We discuss the implications for understanding uncertainty in relation to perceived affordances in the context of political-ideological adherence and affiliation, as well as suggest future research and methodological possibilities investigating dynamic complexity with the survey method.

Basic tests were carried out to determine the baseline effect of the scenario and if the dynamic narrative portion was necessary in the first place (i.e., produced responses that varied from the baseline effect). Base tendencies to invite and report the family indicated that, at whole group level, the scenario leads to inviting the family to the pot luck and not reporting to the neighborhood watch group. These two initial response items are completed before participants are exposed to the added situations, and are in this context representative of a more ’static’ or cross-sectional measure. For our sample the variation of the responses in the dynamic narrative portion showed that as the situations changed, so did the percentage of participants willing to stop inviting and start reporting. We draw the conclusion that for the two affordance measures, adding the dynamic portion does indeed show us something beyond the initial, static measure. Additionally, when dividing the percentage of participants who invite and report on cluster, an even clearer trend emerges. Cluster 1 (low adherence to ideologies, left political leaning) continue to almost exclusively invite the family to the potluck and not report to the neighborhood watch group. Most of the variability in stopping to invite and starting to report is found for Cluster 2 and 3 as uncomfortableness is increased (and vice versa when decreased). Our conclusion is that while our and previous research has found higher ideological constraint on behavior for more left leaning political affiliation, the behavioral pattern of more right leaning participants is still predictable. That is, more right leaning individuals are also constrained in ways aligned with ideology -just one that requires a dynamic framework to emerge.

### 4.1. Hypothesis 1: Relations Between Clusters on Political Ideologies and Affiliation

Three main findings can be deduced from the cluster analysis and results from testing Hypothesis 1. The first one is that there is a main division between Cluster 1 and Cluster 2 and 3 on adherence to all the ideological scales and Political Affiliation. Cluster 1 participants are skewed left on the Political Affiliation scale with most participants choosing “Progressive” or “Liberal” that best approximates their political leaning. The finding that low adherence to the three ideological scales also cluster with left-leaning political affiliation is in line with previous literature [100] that has also found a polarising effect of policy decisions in the context of perceptions of immigrants [101]. In fact, the effect sizes show a strong fit with clustering explaining 37% of variation in political affiliation, 50 and 57% respectively for neoliberalism and ethnocentrism, and 75% in Islamophobia. This shows that the current study setup collectively replicates findings in studies where different subsets of the scales have been used. This should be considered validating in the context of having a relatively smaller sample size than is common in the field. Analyzing the same data with two clusters places only four more individuals in Cluster 1 and the rest in a combined cluster, which makes sense considering the large differences between Cluster 1 and Cluster 2 and 3, as well as that Cluster 2 and 3 are not significantly different on the neoliberal, ethnocentrism, or Political Affiliation scale. Our selection to use three clusters however was more reliant on the finding that Cluster 2 and 3 differed on Islamophobia even if they did not on the other scales or political affiliation. We believe it is an interesting aspect that in our sample we do not see a differentiation between the number of people choosing Liberal political affiliation compared to Conservative despite the ideological difference, since it supports the idea that liberalism is sometimes only a facade without deeper commitments to for example anti-racism [102]. The conclusion here is that ideological constraint does not cleanly line up with political affiliation unless one aligns with an affiliation more left than Liberal.

### 4.2. Hypothesis 2: The Relation Between Clustering and Uncertainty

From testing the second hypothesis the main result is that Cluster 1 is close to *H* = 0 on uncertainty as it applies to change in response in invitability and slightly higher but still low (*H* = 0.2) in reportability. Both of these are significantly different to Cluster 2 and 3, which are not significantly different from each other on either affordance. Despite the significant difference in Islamophobia, the lower reported Islamophobia in Cluster 2 (compared to Cluster 3) still entails higher uncertainty in change in response to the dynamic narrative items. Coupling this to the finding that Cluster 2 and 3 stop inviting and start reporting (see Figure 2) relates the content to what it is that (Shannon information) uncertainty is describing: from an onlooker’s perspective participants belonging to Cluster 2 and 3 waver on their reported inclusivity, something that presumably affects how much one would be trusted by others to be included and not reported. Higher uncertainty in Cluster 2 and 3 means that knowing if someone has stopped inviting/started reporting gives little information as to whether they in the next situation will change their behavior. In our sample, uncertainty is statistically coupled to adherence to ideologies and Political Affiliation, which then also can act as indicators for if a change (or non-change) in behavior provides information about what that person will do in the next situation. In real life then, while knowing if someone is a Progressive could lead to less uncertainty about their future behaviors, differentiating between Liberals and Conservatives does not change the amount of uncertainty when observing theirs. In our case therefore, being constrained by ideological commitments means that regardless of comfortability, Progressive and more left leaning individuals keep inviting and not reporting the family. One instance of observing such a person’s behavior is representative of their overall behavioral pattern. In real life this translates into an ability to trust such people to have one’s best interests in mind. In contrast, even if more right leaning people espouse less Islamophobia, it is more uncertain whether observing one of their behaviors represent their behavioral repertoire and more observations are needed to be able to draw conclusions about them. This should be taken to mean that in the context presented here, Shannon information and uncertainty represents (in common language) potential trust based on limited information.

### 4.3. Limitations and Future Research

This research project combines dynamic and relational methods and analyses of social psychological phenomena. Laboratory and observational methods could also be leveraged to take another step towards more ecologically valid testing [103,104]. Social psychologists may react to the number of participants in the study and the limits on generalization that this entails. We have been careful here not to generalize too far beyond the relationships between variables in our study and what our sample reported. However, it should also be mentioned that population-generalizations need not be the goal even in a more mainstream psychological research project [27]. For example, within the clusters in our sample there are signs that reality is still more complex, and responses more idiosynchratic, than what we have been able to report here: one participant in Cluster 1 had a (relatively) high uncertainty value, and a few individuals in Cluster 2 and 3 had (relatively) low ones. One of the important contributions of a research project such as this one is to demonstrate the varied kinds of dynamic patterns that exist and understand if there are generalizable patterns and processes. Of course, for generalization purposes it would still be interesting to carry out this research on a much larger sample as it opens up for even more nuance and variability to aid our understanding of the phenomena at hand.

Another limitation is the constrained use of the concept of affordances from Ecological Psychology [10]: invitability and reportability were chosen due to that they can be connected to real actions. We also suspected that asking about behaviors might lead to less social desirability bias [105] something that can be supported by the difference between Cluster 2 and 3 on Islamophobia (which is a more explicit measure) but which is not carried through into reported affordances. However, the use of affordances here is still a ‘question on a page’ in the survey and not actually acted upon or measured in a real situation, something that has been shown to make a difference in how people respond to experimental contexts [106] (although the ethical dimension would need deliberation). In relation to this, we also use three traditional scales that are measured statically, justified here through the assumption that although these measures may be dynamic over a longer time frame, that during the time-span of the experiment they can be assumed to be approximately static. Also, while using a conception of ideology as global constraint on behavior, how we measure it here becomes a limitation because it follows (at least in part) a reductionist ontology, that ideologies are static traits measurable by combining additive questions about similar groupings of behaviors. To remedy this in future research, the difficult task of developing alternative ways of measuring ideologies is needed. Lastly, we encourage the possibility of exploring people’s lived experiences in this context with qualitative methodologies, perhaps in combination with discourse analysis, as it would highlight the real life process of dealing with uncertainty, which possibilities for action that are available, and how we could increase them to reduce uncertainty.

## 5. Conclusions

Applying a dynamic systems framework to the survey method, our study demonstrates a methodological pathway to understanding the impact of ideological constraints on individual response patterns in a controlled setting. Also, it takes a step toward more closely resembling real life settings where information is constantly developing and responses change. Depending on adherence to different ideologies and political leaning, participants’ perception was altered in the dynamic scenario context, affecting their response process. In addition, tied to ideological and political clustering was a relation to uncertainty in response change to both invitability and reportability. Our results show that weaker adherence to the ideologies measured here and more leftist political leaning is related to less uncertainty about being included and not excluded, even in the context of changing comfortableness in (otherwise benign) situations.

More research tying human behavior to complexity is needed as it works as a counter-weight to the (often dichotomous) over-simplifications that neoliberalism and related ideologies offer. Neoliberalism uniquely unites us globally and is well-accepted by scholars to have become hegemonic and incorporated into the common-sense ways we live in and understand our world [36,37,38]. Neoliberalism’s pervasiveness lends itself to an integral role in Western culture in shaping our perceptions of those who are lesser known such as the immigrants who move in next door, the people with the indiscernible accents, and the neighbors living in ways we don’t recognize. Islamophobia and ethnocentrism, which support and are magnified by neoliberal values, also largely define Western policy and culture, and have become embedded in our everyday responses and perceptions. While neoliberalism, Islamophobia, and ethnocentrism are distinct in their nature and scope, they contribute to certain similar impacts such as the moral exclusion of migrants of Color, particularly those associated with Islam.

## Figures and Tables

**Table 1:** Unstandardized Mean (and Standard Deviation) for each Cluster by Ideological Scale and Political Affiliation.

	Neoliberalism	Islamophobia	Ethnocentrism	Political Affiliation
Cluster 1	49 (13)	31 (10)	32 (7)	2.3 (1.6)
Cluster 2	86 (15)	52 (16)	48 (8)	5.6 (2.2)
Cluster 3	87 (17)	111 (26)	51 (9)	5.6 (2.4)

## Data Availability

The data for this study is not freely available due to Swedish ethics legislation and can only be provided after an amendment to the Swedish IRB protocol.

## Abbreviations

The following abbreviations are used in this manuscript:

ANOVA	Analysis of Variance
CI	Confidence Interval
US	United States (of America)

## Appendix B

The twelve situations making up the dynamic narrative portion: Series 1, comfortable-to-neutral-to-uncomfortable.

1. Comfortable: While watering your lawn outside, you see the new neighbors. You strike up a conversation with the mother and find out that they have two kids, her spouse is an engineer, and she works from home.
2. Comfortable: A couple of days later, while walking to a neighborhood activity, you see the kids outside playing hopscotch and laughing with some older kids while they’re playing word games.
3. Neutral: The next day, you see the kids playing outside and in your conversation with them, they mention they’re from Syria. A few moments later the mother rushes out of the house and hurries the kids in, barely making eye contact with you.
4. Neutral: After another couple of days, you notice that your new neighbors never leave the area, despite them having a car parked outside. You recall that the father might be an engineer and wonder if he hasn’t started work yet.
5. Uncomfortable: For the last day or two, you’ve noticed how the trash bins outside of the house are overflowing and starting to smell.
6. Uncomfortable: On a walk with Nicole, the neighborhood gossip, she mentioned that she saw the new neighbors using the community center resources and community pool. However, Nicole continued, she doesn’t think they are documented and are not paying taxes for those public services.

Series 2, negative-to-neutral-to-positive.

7. Uncomfortable: On a late night walk around the block, you hear adults and children yelling from the new neighbor’s house.
8. Uncomfortable: The next day you speak with Nicole, she says someone should have called the police last night because of the disturbance from the neighbor’s house. Nicole thinks that your neighbors must not be good parents.
9. Neutral: Later that day you see the new neighbor’s kids playing outside again and they stop to talk to you. The kids mention that their cousin in Syria had a baby last night and everyone was yelling with excitement about the news. After your conversation, they say they need to go back inside to pray.
10. Neutral: A few days later while outside, you notice the new neighbor’s windows are open wide and you see the kids cleaning up and doing chores.
11. Comfortable: A short while later, the father comes out of the house and you notice the smell of delicious food and you ask about it. The father offers some to you.
12. Comfortable: The next day, you see the mother again and strike up a conversation, she mentions that she worked for the US government as a translator and you see her kids run out of the house and give her a hug.
